# Declined contrast sensitivity of neurons along the visual pathway in aging cats

**DOI:** 10.3389/fnagi.2014.00163

**Published:** 2014-07-09

**Authors:** Zhengchun Wang, Zhimo Yao, Nini Yuan, Zhen Liang, Guangxing Li, Yifeng Zhou

**Affiliations:** ^1^Hefei National Laboratory for Physical Sciences at the Microscale and School of Life Sciences, University of Science and Technology of ChinaHefei, Anhui, China; ^2^Department of Bio-Medical Engineering and School of Life Sciences, Anhui Medical UniversityHefei, Anhui, China; ^3^McGill Vision Research, McGill UniversityMontreal, Quebec, Canada

**Keywords:** aging, cat, contrast sensitivity, degeneration, visual pathway

## Abstract

Changes in the visual cortex appear to mediate much of the visual degradation during normal aging. However, how aging affects different stages along the visual pathway is unclear. In the current study, the contrast response function, one of the most important properties of neurons from early visual areas to high brain areas, was systematically compared along the visual pathway, including the lateral geniculate nucleus (LGN), early visual cortices (A17 and A18), and posteromedial lateral suprasylvian cortex (PMLS, analog to the medial temporal area (MT) in monkeys) of young and old cats. We found that the effects of aging on the LGN were negligible, whereas those in the striate cortex were substantial, with even more severe degradation in the PMLS. Reduced contrast sensitivity of neurons in the three cortical areas was accompanied by enhanced maximal visual response, increased spontaneous activity, and decreased signal-to-noise ratio, while LGN neurons exhibited largely normal response properties. Our results suggested that there was a progressively greater effect of aging on neurons at successively higher stages in the visual pathway.

## Introduction

Senescence is associated with a decline in many aspects of visual functions (Baracat and Marquie, 1992; Schefrin et al., 1999; Bennett et al., 2007). Notably, the age-related degeneration of perception of higher-order stimuli (e.g., contrast-defined second-order patterns) is significantly more pronounced, and can be detected much earlier than lower-order tasks (e.g., luminance-defined first-order stimuli) (Habak and Faubert, 2000; Tang and Zhou, 2009). These impairments cannot be solely due to optical changes or changes in the retina alone (Elliott et al., 1990; Owsley, 2011), and probably reflect age-related alterations occurring in the central nervous system, i.e., postretinal visual information processing (Higgins et al., 1988; Spear, 1993; Schmolesky et al., 2000).

Several studies have provided evidence for a degeneration of neural response properties in the visual cortex of various senescent animal models (Schmolesky et al., 2000; Hua et al., 2006; Liang et al., 2010; Fu et al., 2013), which laid the groundwork for studies of the neurobiological mechanisms of such age-related effects. Specifically, the findings on senescent rhesus monkeys (Schmolesky et al., 2000), aged cats (Hua et al., 2006), and aged rats (Wang et al., 2006) suggested that the age-related changes of cortical function in different mammalian species with well-developed cortices were similar. Previous work from our lab suggested that the degeneration between visual areas was different (Wang et al., 2005; Yu et al., 2006; Yang et al., 2009). However, these independent studies, each focusing only on one or two visual areas, failed to provide a better understanding of the aging process along the entire visual pathway. Additionally, attempts at cross-study comparisons are difficult, due to the different experimental conditions (e.g., stimulus patterns and protocols) used in these studies. Thus, previous studies fell short of an effective assessment of hierarchical aggravation, such as a systematic, progressive increase of impairment along the visual pathway. Examination of a series of brain areas along the visual pathway in the same experimental conditions would provide a more accurate picture of age-related changes.

In the current study, we examined how neurons at different stages in the visual pathway of cats responded to variations in the contrast of sine wave grating stimuli. Specifically, we compared contrast responses of neurons in lateral geniculate nucleus (LGN), early visual cortices (A17 and A18), and posteromedial lateral suprasylvian cortex (PMLS) in young and old cats, using the same experimental conditions, because the contrast coding has been demonstrated to exist at different visual stages (Movshon et al., 1978a; Sclar et al., 1990; Ledgeway et al., 2005). The animal model used in the current study was the cat, which has been demonstrated to be a good model to study aging effects on the visual system (Hua et al., 2006, 2010).

## Material and methods

### Subjects

Experiments were conducted on six young adult cats (1 to 3 years of age) and four old cats (11 to 13 years of age). Several lines of evidences indicated that a 12-months-old cat can be considered sexually mature, and that functional aging of the brain takes place in cats of 10 years or older (Bowersox et al., 1984a,b; Harrison and Buchwald, 1985; Levine et al., 1986).

All cats were examined ophthalmoscopically and by retinoscopy prior to recording. No visible deteriorations in the eyes occurred during the experimental period in either old or young animals. All procedures were approved by the Animal Care and Use Committee of the University of Science and Technology of China and in accordance with the National Institutes of Health Guide for the Care and Use of Laboratory Animals.

### Preparation for extracellular recording

Animals were initially anesthetized with ketamine HCl (20 mg/kg, i.m.) for venous cannulation, followed by propofol (6 mg/kg/h, i.v.) during subsequent surgery. The level of anesthesia was assessed by periodically applying nociceptive stimulation (toe pinch), and monitoring the heart rate and ECG (Mindray, PM-7000). All pressure points were treated with lidocaine-HCl jelly (2%), and surgical wounds were treated with bupivacaine (0.25%). After venous cannulation and tracheotomy, the animal was mounted in a stereotaxic apparatus. Anesthesia was maintained with a continuous infusion of propofol (5 mg/kg/h, i.v.) and sufentanil (10 ng/kg/h, i.v.). Craniotomy was performed above the area in the PMLS, A18, A17, and LGN (see below for details). After surgical procedures were finished and a stable and good physical state of the anesthetized animal was assured, the animal was then paralyzed with Gallamine triethiodide (loading dose, 8 mg/kg, followed by 10 mg/kg/h, i.v.). During recording, animals were artificially ventilated, and end tidal CO_2_ was monitored and maintained at approximately 3.8−4.3%. Rectal temperature was maintained around 37.5°C by an automatically regulated heating pad. Throughout the remainder of the experiment, all vital signs were monitored and maintained at normal levels. Penicillin (200000 U, i.m.), dexamethasone (5 mg, i.m.), and atropine (1 mg, i.m.) were administered every 12 h, throughout the experiment.

The corneas were initially protected with topical carboxymethylcellulose (1%). Neosynephrine (5%) and atropine sulfate (1%) were topically administered to retract nictitating membranes and dilate pupils, respectively. Zero power air permeable contact lenses were fitted to protect the corneas. Corrected spectacle lenses were used as needed during recording. The optic disc of the eye was back-projected on a tangent screen positioned 57 cm from the retina (Fernald and Chase, 1971) and the area centralis of the eye was located (Nikara et al., 1968).

Epoxy coated, high impedance (2−5 MΩ) tungsten electrodes (Frederick Haer & Co (FHC)) were positioned and advanced using a hydraulic micromanipulator (David Kopf Instruments, Tujunga, California, USA). For PMLS, a 3 mm craniotomy was made at H-C coordinates A2/L13-21. The electrode was advanced through a dural incision at an angle of 30° perpendicular to the bank of the suprasylvian sulcus (Vajda et al., 2004). For A18, a 3 mm craniotomy was centered at H-C coordinates A3/L4. For A17, a 3 mm craniotomy was centered at H-C coordinates P4/L1. For both areas, A17 and A18, the electrodes were advanced vertically. For LGN, a 3 mm craniotomy was centered at H-C coordinates A6/L9 (Bishop et al., 1962). Agar (4% in saline), sealed with petroleum jelly, was applied to protect the dura. Recordings were, in turn, made in the PMLS, A18, A17, and LGN to avoid detrimental effects on higher hierarchical areas caused by penetrations made in lower ones.

### Visual stimulus

Stimuli were drifting sine wave gratings displayed on a gamma-corrected 19 inch CRT monitor (1024 × 768, 85 Hz, Sony, G220), placed 57 cm in front of the eyes. The mean luminance of the display was 45.2 cd/m^2^. The program to generate stimuli was written in MATLAB (The MathWorks, Natick, MA, USA), using extensions provided by the Psychophysics Toolbox (Brainard, 1997; Pelli, 1997). Grating contrast was quantified as the difference between the maximal and minimal luminance divided by their sum (Michelson contrast). The optimal orientation, spatial frequency, and temporal frequency of each neuron were determined with conventional tuning curve experiments. The optimal position and size of the grating were determined through quantitatively mapping the receptive field with small grating patches and size tuning curves, respectively. All gratings were subsequently presented with optimal stimulus parameters to the neuron’s dominant eye. For each trial, a blank screen of mean luminance was first presented for 1 s, followed by drifting gratings of different contrasts (14 levels ranging from 0.0 to 0.99), and finally a blank screen period of 0.5 s. To eliminate effects of adaptation when measuring contrast response functions, all stimuli were presented in a pseudo-random order.

### Data collection and analysis

Signals were filtered (300 Hz–3 kHz) and amplified (×10 k, Dagan 2400A). Action potentials were firstly fed into a window discriminator with an audio monitor (Winston Electronics, St. Louis, MO, USA) and then digitized (40 kHz) using a data acquisition board (National Instruments, Austin, TX, USA) controlled by IGOR software (WaveMetrics, Lake Oswego, OR, USA), and finally saved for off-line analysis. Contrast response functions (CRFs) were constructed as the mean spike rate plotted as a function of stimulus contrast. Spontaneous activity (baseline firing rate) was measured as the mean response to a blank screen (contrast value of zero). A neuron’s signal-to-noise ratio (SNR) was defined as the ratio of the maximal attainable response and its spontaneous activity (Schmolesky et al., 2000; Leventhal et al., 2003). Spontaneous activity values below 1 spike/s were set equal to 1 spike/s for the SNR analyses (Schmolesky et al., 2000).

The CRF of each cell was fitted with a Naka-Rushton equation (Albrecht and Hamilton, 1982; Albrecht, 1995):

(1) $$R=R_{\max}C^{n}/(C^{n}+C_{50}^{n})+M;$$

where *R* is the neuron’s response to a contrast value of *C*. *C*_50_, *R*_max_, *n*, and *M* denote the contrast at which the response reaches half of its maximal value, the maximal attainable response, the steepness of the curve, and the spontaneous activity, respectively. Unlike psychophysical experiments in which contrast sensitivity is defined as the reciprocal of the lowest detectable contrast, *C*_50_ has been demonstrated to be a reliable index in electrophysiology experiments (Albrecht and Hamilton, 1982; Sclar et al., 1990; Yang et al., 2008). Smaller values of *C*_50_ corresponded to higher contrast sensitivity of neurons.

Many LGN cells did not saturate with the increase in stimulus contrast, and therefore had response functions that could not be properly fit with the Naka-Rushton function. For those cells, we estimated half *R_max_* contrast (*C*_H_) values to assess contrast sensitivity. Specifically, the raw *R*_max_, defined as the maximal evoked response, was obtained by counting the numbers of the spikes, and then dividing by two, to yield the half *R*_max_ values (*R*_H_). We traced the *R*_H_ value in the raw data and found the two adjacent points lower than *R*_H_ and the two adjacent points higher than *R*_H_. *C*_H_ was determined by fitting the four points with a linear equation:

(2) $$R-R_{\text{H}}=k_{\text{H}}\left(C-C_{\text{H}}\right);$$

where *R_H_* denotes the response that is half of the maximal response. The coefficient *k*_H_ describes the slope of the line; *C*_H_ is the corresponding contrast that induces *R*_H_. Here, *C*_H_ was used as the index of contrast sensitivity of each neuron. Smaller *C*_H_ values corresponded to greater contrast sensitivity of neurons.

## Results

A total of 195 neurons from six young adult cats and 166 neurons from four old cats were studied. Specifically, we recorded 38 LGN cells, 69 A17 cells, 49 A18 cells, and 39 PMLS cells in young adult cats, and 36 LGN cells, 34 A17 cells, 52 A18 cells, and 44 PMLS cells in old cats. All data were collected from two to four penetrations in each visual area. Neurons recorded from each group of cats were at the same range of depth from the pial surface of the brain, representing random samples of neurons in all cortical layers. All cells had receptive fields within 25 degrees of the area centralis and most were within 10 degrees (>90% for all studied visual areas). No significant difference was found in the eccentricity distribution of neurons in these four brain areas between the young and old groups (*P* > 0.2 for all brain areas, *t*-test). Subjects for this study are abbreviated as OC (old cats) and YC (young adult cats) in tables and figures.

### Effect of aging on LGN neurons

**Figure 1:**
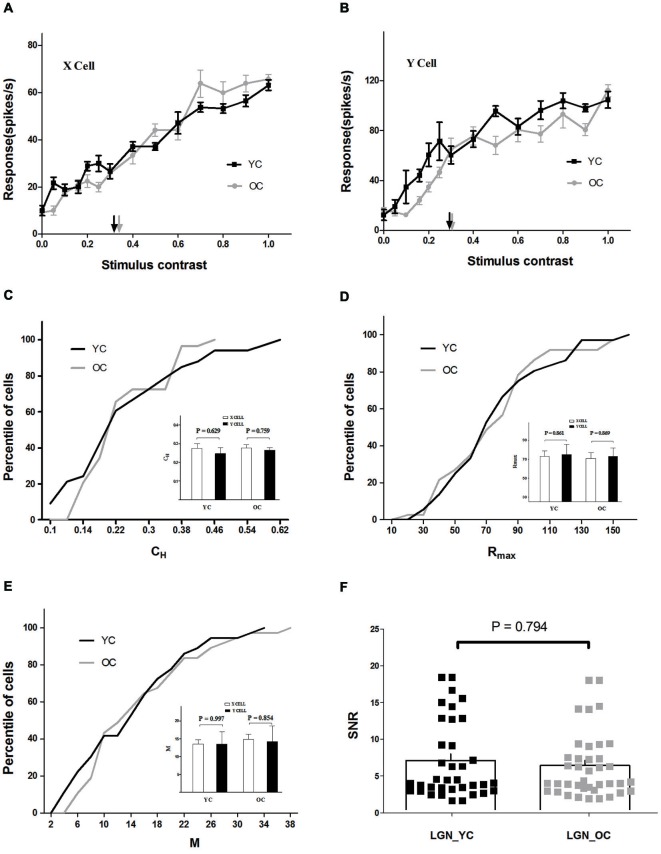
**Contrast response properties of lateral geniculate nucleus (LGN) neurons in young and old cats. (A)** Contrast response functions of typical young cats (YC) (black) and old cats (OC) (gray) X cells. Each point represents the response to the stimulus at a given contrast. The half maximal contrast (*C_H_*) is marked with black (YC) and gray (OC) arrows. **(B)** Contrast response functions of typical young (black) and old (gray) Y cells in the same format as **(A)**. The population results of *C*_H_, *R*_max_, and *M* values are illustrated in **C**, **D**, and **E**. Comparison of each parameter between X and Y cells, in young and old cats, is shown on the inset plots in **C**, **D**, and **E**. Black and gray lines represent the data which combined X and Y cells of young and old cats, respectively. **(F)** Distribution of signal-to-noise ratio (SNR) values in LGN for young (black squares) and old (gray squares) cats. The histograms indicate the mean value of SNR values in each group. Error bars indicate SEM. The same abbreviations are used in subsequent figures.

A substantial proportion of cells in LGN (>91% for both young and old groups) did not saturate with increasing contrast. There was no plateau in the CRF (Figures 1A,B), which could not be fit with a Naka-Rushton function. Alternatively, we estimated contrast sensitivity with *C_H_* (See Materials and Methods).

Cells in LGN were classified as X cells and Y cells (Hochstein and Shapley, 1976). The average *C_H_* values of X and Y cells in young and old cats are shown in an inset plot of Figure 1C. Y cells exhibited smaller *C*_H_ values than X cells in both young (0.25 ± 0.03 for Y cells, 0.27 ± 0.03 for X cells) and old (0.26 ± 0.01 for Y cells, 0.28 ± 0.02 for X cells) groups, while there was no significant difference between X and Y cells (*P* = 0.629 for young cats, *P* = 0.759 for old cats, *t*-test).

There was also no significant difference between *R_max_* values of X and Y cells (*P* = 0.861 for young cats, *P* = 0.869 for old cats, *t*-test), although X cells, on average, exhibited smaller *R*_max_ values than Y cells in both young (73.01 ± 5.76 for X cells, 75.20 ± 10.52 for Y cells) and old (70.76 ± 6.02 for X cells, 72.93 ± 9.08 for Y cells) groups (inset plot, Figure 1D). Additionally, the average *M* values of X were almost identical to those of Y cells in both young (13.43 ± 1.27 for X cells, 13.44 ± 3.56 for Y cells) and old (14.82 ± 1.35 for X cells, 14.17 ± 4.44 for Y cells) cats (inset plot, Figure 1E), and there was no significant difference between X and Y cells (*P* = 0.997 for young cats, *P* = 0.854 for old cats, *t*-test). Because no differential effect of aging on the X and Y cells was observed, data from these two types of cells were combined for convenience and are shown in Table 1 and the analyses thereafter.

**Table 1:** **Visual response properties of each brain area for YC and OC groups**.

		**LGN**	**A17**	**A18**	**PMLS**
**N**	**YC**	38	69	49	39
	**OC**	36	34	52	44
***C*_50_**	**YC**	0.27 ± 0.03(*C_H_*)	0.25 ± 0.01	0.24 ± 0.01	0.19 ± 0.01
	**OC**	0.27 ± 0.02 (*C_H_*)	0.33 ± 0.02	0.33 ± 0.01	0.33 ± 0.01
***R*_max_**	**YC**	73.48 ± 5.27	51.82 ± 2.71	55.09 ± 2.96	55.07 ± 4.05
	**OC**	71.19 ± 4.97	62.32 ± 4.67	74.93 ± 3.10	68.99 ± 4.74
***M***	**YC**	13.73 ± 1.37	4.98 ± 0.34	6.16 ± 0.50	4.49 ± 0.39
	**OC**	14.70 ± 1.32	6.71 ± 0.67	8.30 ± 0.65	7.46 ± 0.75
***n***	**YC**	117.63 ± 18.92(*k_H_*)	2.70 ± 0.14	2.77 ± 0.17	2.44 ± 0.16
	**OC**	137.12 ± 16.94(*k_H_*)	2.60 ± 0.22	2. 70 ± 0.11	2.10 ± 0.12
**SNR**	**YC**	6.01 ± 0.47	15.32 ± 1.30	15.47 ± 1.94	16.79 ± 2.38
	**OC**	5.97 ± 0.40	11.80 ± 1.43	9.56 ± 0.58	11.24 ± 1.24

*Each data row, from top to bottom, represents the number of cells (N), the contrast at which the response reaches half of its maximal value (C_50_), the maximal response (R_max_), the spontaneous activity (M), the slope of the Naka-Rushton fit (*n*), and the signal-to-noise ratio (SNR) for each visual area. For LGN, the values of half maximal contrast (C_H_) and the steepness of the line (k_H_) are displayed. All values (except for N) are expressed as mean ± SEM. YC = young cats, OC = old cats, LGN = lateral geniculate nucleus, PMLS = posteromedial lateral suprasylvian cortex*.

Results demonstrated that *C*_H_ and other response properties of LGN neurons in old cats were relatively unchanged compared with young ones (Table 1 and Figure 1). The percentile of LGN neurons with given *C*_H_, *R*_max_, and *M* values did not display significant differences between young and old neurons (*P* = 0.548, *P* = 0.858, and *P* = 0.574, respectively, *t*-test). Furthermore, there was no significant difference between SNR values of young (6.01 ± 0.47) and old neurons (5.97 ± 0.40; *P* = 0.794, *t*-test) in LGN (Figure 1F).

The present results indicated that there was no significant age-related impairment in area LGN. The later brain areas in the visual pathway were examined subsequently in young and old cats.

### Effect of aging on the A17 and A18 neurons of the early visual cortices

Cells in A17 and A18 were classified as simple and complex cells (Movshon and Tolhurst, 1975; Skottun et al., 1991). For A17 cells, the *C*_50_, *R*_max_, *M*, and *n* values of simple cells were similar to those of complex cells in the young (*P* = 0.921 for *C*_50_, *P* = 0.757 for *R*_max_, *P* = 0.989 for *M*, and *P* = 0.927 for *n*, *t*-test) and old cats (*P* = 0.806 for *C*_50_, *P* = 0.898 for *R*_max_, *P* = 0.806 for *M*, and *P* = 0.865 for *n*, *t*-test). For A18 cells, the *C*_50_, *R*_max_, *M*, and *n* values of simple cells and those of complex cells had no significant difference, either in the young group (*P* = 0.643 for *C*_50_, *P* = 0.412 for *R*_max_, *P* = 0.145 for *M*, and *P* = 0.922 for *n*, *t*-test) or in the old group (*P* = 0.605 for *C*_50_, *P* = 0.980 for *R*_max_, *P* = 0.650 for *M*, and *P* = 0.818 for *n*, *t*-test). Because simple and complex cells exhibited similar age-related changes, data from these two types of cells were combined.

**Figure 2:**
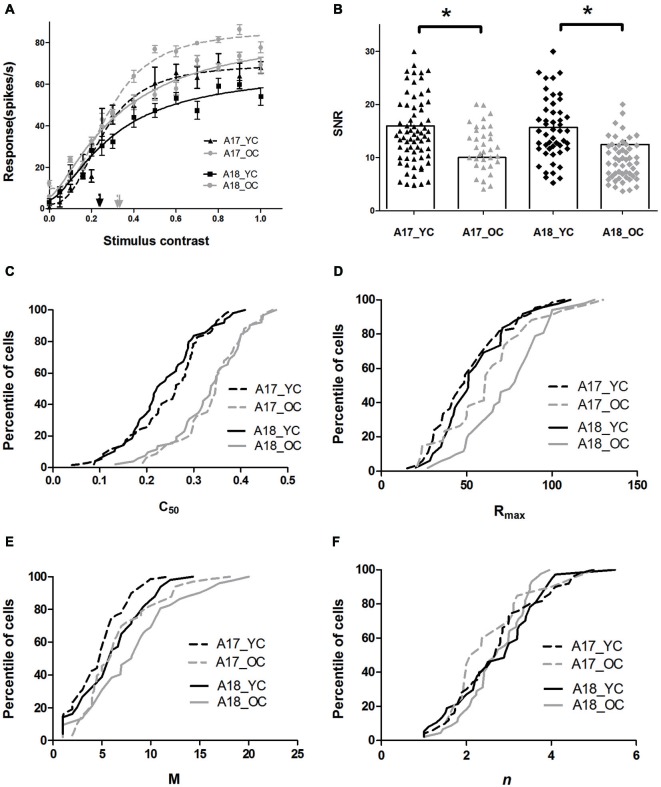
**Contrast response properties of A17 and A18 neurons in young and old cats. (A)** Example curve fit for contrast response functions (CRFs) of typical young neurons (black line) and typical old neurons (gray line) recorded from A17 (dotted lines) and A18 (solid lines) neurons. Each point represents the response to the stimulus at a given contrast. The fitted curves take the form of a Naka-Rushton function with the *C*_50_ values marked with black (YC) and gray (OC) arrows. **(B)** Distribution of SNR values in areas A17 (black triangles for young cats, gray triangles for old cats), and A18 (black diamonds for young cats, gray diamonds for old cats), where histograms indicate mean values of SNR values in each group. The population results for fitted parameter values in A17 (dotted lines) and A18 (solid lines) are illustrated in panels **2C–F**. The black and gray lines represent the data of young and old cats, respectively. Error bars indicate SEM.

The results demonstrated that both A17 and A18 neurons, in old cats, exhibited significantly increased *C*_50_ values, elevated maximal visual response and spontaneous activity, and decreased SNR values when compared with young ones. Figure 2A illustrates examples of curve fits obtained from young (black lines) and old (gray lines) cats in A17 and A18 neurons. Four features of these curves are evident in Figure 2A: (1) the *C*_50_ values of neurons in old cats, both A17 and A18, are larger than observed in young cats; (2) both A17 and A18 neurons in old cats have greater *R*_max_ values than young ones, i.e., they saturate at higher response levels than the young ones; (3) the old neurons have greater *M* values than the young ones, i.e., higher spontaneous activity of old neurons than young ones; and; (4) the steepness of the curves, for both A17 and A18 neurons, have no significant changes between young and old, i.e., similar *n* values.

The population results for fitted parameter values are illustrated in Figures 2C–F. Both A17 and A18 neurons in young cats had smaller *C*_50_ values (0.25 ± 0.01 for A17; 0.24 ± 0.01 for A18) than neurons in old cats (0.33 ± 0.02 for A17; 0.33 ± 0.01 for A18) (*P* < 0.001 for both areas, *t*-test). For *R*_max_ values in A17 and A18 neurons, there were significant increases in old neurons compared with the young neurons (*P* < 0.05 for A17 and *P* < 0.001 for A18, *t*-test). There were also significant increases in *M* values, both in A17 and A18 neurons of old cats, when compared with the young ones (*P* < 0.05 for both areas, *t*-test). However, the slopes of the CRF (*n*), for both A17 and A18, had no significant changes between young and old cats (*P* = 0.691 for A17; *P* = 0.744 for A18, *t*-test; Table 1 and Figure 2F).

Spontaneous activity values, for both A17 and A18 in old cats, were enhanced much more than the maximal visual responses, resulting in significantly decreased SNRs (Table 1 and Figure 2B). Old neurons displayed smaller SNR values (11.80 ± 1.43 for A17; 9.56 ± 0.58 for A18) when compared with young neurons (15.32 ± 1.30 for A17; 15.47 ± 1.94 for A18, *P* < 0.05 for both areas, *t*-test). Most cells (63.8%) in A17 of young cats had SNR values higher than 12, while only 44.1% of the neurons in old cats had SNR values more than 12. Similar results were obtained from neurons in A18. Most cells (71.3%) in A18 of young cats had SNR values higher than 12, while only 26.4% of the neurons in old cats had SNR values more than 12. Reduced SNR values for old neurons in A17 and A18 suggested an impaired ability to convey signals from a noisy background.

From what has been described above, we have shown a detrimental effect of aging on contrast response properties of neurons in A17 and A18. To estimate the possible changes at later stages in the visual processing during normal aging, the PMLS was also examined in young and old cats.

### Effect of aging on PMLS neurons

**Figure 3:**
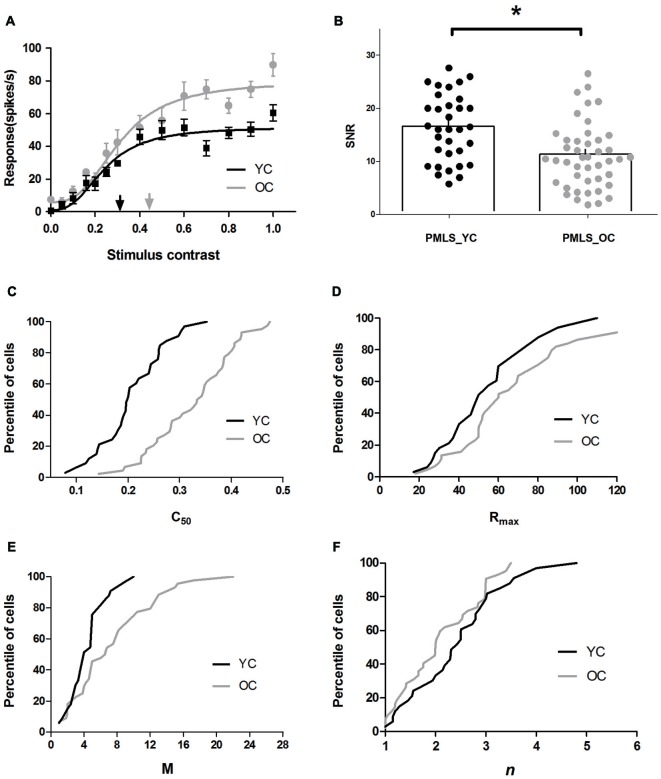
**Contrast response properties of posteromedial lateral suprasylvian cortex (PMLS) neurons in young and old cats. (A)** Sample CRF curve fits for PMLS neurons in the same format as in Figure 2A. **(B)** Distribution of SNR values in area PMLS for young (black dots) and old (gray dots) cats, and mean values (histogram) of SNR values are illustrated. Percentile of PMLS neurons with given *C*_50_, *R*_max_, *M*, and *n* values are shown in panels **C**, **D**, **E**, and **F**, where black and gray lines represent the data of young and old cats, respectively. Error bars indicate SEM.

The results indicated significantly increased *C*_50_ values, elevated maximal visual responses and spontaneous activity, and decreased SNR of PMLS neurons in old cats when compared to young ones. Figure 3A illustrates representative contrast response data and curve fits for young and old cats. Four features of these curves are evident in Figure 3A: (1) the *C*_50_ value obtained from neurons in old cats was larger than observed in young cats; (2) the aged cells had greater *R*_max_ values than the young cells, i.e., they saturated at a higher response level than the young ones; (3) the old neurons had larger *M* values than the young ones, i.e., a higher spontaneous activity of the old neurons than the young neurons; and; (4) the steepness of the curve was similar between these two groups.

The population results for fitted parameter values are illustrated in Figures 3C–F. It was evident that PMLS neurons in old cats had significantly larger *C*_50_ values (0.33 ± 0.01) than in young adult cats (0.19 ± 0.01; *P* < 0.001, *t*-test; Figure 3C). We also compared the maximal visual response (*R*_max_) and spontaneous activity (*M*) of PMLS neurons between young and old cats. Both *R*_max_ and *M* values increased (*P* < 0.05 for both parameters, *t*-test) in old neurons compared with the young ones (Table 1, Figures 3D,E). However, the slope of the CRF (*n*) had no statistically significant change between young and old cats (*P* = 0.149, *t*-test; Table 1 and Figure 3F).

We also found that spontaneous activity was affected greatly by aging, but the maximal visual response was only moderately affected. In general, old cat neurons increased their *M* values by 66.1%, while the *R*_max_ values increased only by 25.3%, which resulted in sizable decreases in their SNR values. The average SNR for old cats (11.24 ± 1.24) versus young cats (16.79 ± 2.38) displayed a significant decline (*P* < 0.05, *t*-test; Table 1, Figure 3B). Only 38.6% of the neurons in old cats had ratios more than 12, while in young cats, most cells (69. 7%) had values higher than 12. The reduced SNR values suggested an impaired ability to convey signals from a noisy background, for old neurons in PMLS.

### Comparison of age-related degeneration of the visual areas

The visual contrast sensitivity of human subjects significantly declines during senescence (Higgins et al., 1988; Santos et al., 2006). To our knowledge, psychophysical contrast sensitivity has not yet been measured on aging cat models, while our previous studies on young adult cats (Hua et al., 2010) demonstrated that the perceptual contrast sensitivity was significantly correlated with the average neuronal contrast sensitivity in cats. We calculated the differences of mean *C_H_* values (Δ*C*_H_) in LGN neurons, and the differences of mean *C*_50_ values (Δ*C*_50_) in A17, A18, and PMLS between young and old neurons. Area PMLS had the largest difference (Δ*C*_50_ = 0.142), followed by areas of A18 and A17, (Δ*C*_50_ = 0.096 and 0.093, respectively), while the Δ*C*_H_ in LGN was negligible. Thus, the higher visual areas apparently exhibited much more severe deterioration than lower ones.

**Figure 4:**
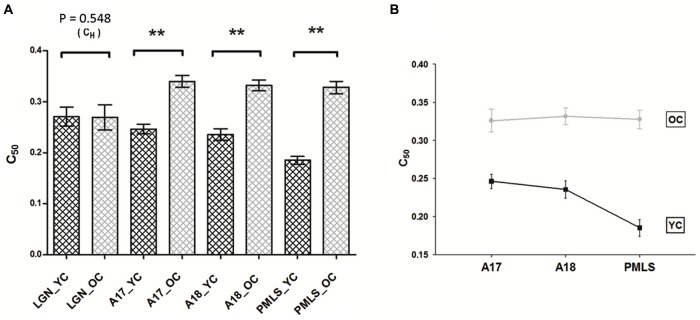
**Comparison of aged-related changes in *C_H_* and *C*_50_ values between LGN, A17, A18, and PMLS. (A)** Aging resulted in significant decline in *C*_50_ values in three cortical areas, but not in LGN. Histograms indicate mean values of *C*_H_ (LGN) and *C*_50_ (for A17, A18, and PMLS) values. **(B)** The general trend of the difference in the three cortical areas. The relative magnitude of *C*_50_ difference in PMLS is greatest, followed by A18 and A17. Error bars indicate SEM. * *P* < 0.05, ** *P* < 0.01.

The results described above were confirmed by a two-way ANOVA to the *C*_50_ values with age (young and old) and brain areas (A17, A18 and PMLS) as group factors. The results revealed a significant effect of age (*P* < 0.001) and brain areas (*P* < 0.001) on *C*_50_ values. This indicated that A17, A18, and PMLS cells in old cats were all less contrast sensitive than those in young cats. More importantly, there was a significant interaction between the effects of age and brain areas on *C*_50_ values (*P* < 0.05) (Figure 4A). Simple effect analyses showed that the *C*_50_ values of PMLS neurons in old cats degenerated significantly more than neurons in A17 (*P* < 0.01). The *C*_50_ values of PMLS neurons in old cats degenerated marginally compared with neurons in A18 (*P* = 0.055). However, the *C*_50_ values of A17 neurons in old cats did not exhibit significant degeneration compared with neurons in A18 (*P* = 0.225; Figure 4B).

We also applied the two-way ANOVA to maximal attained response (*R_max_*) and baseline activity (*M*) values, respectively. The results revealed a significant effect of age (*P* < 0.001 for both parameters). However, there was no interaction of age and brain areas (*P* = 0.403 for *R*_max_, *P* = 0.567 for *M*).

## Discussion

The current study systematically analyzed how the CRFs of neurons, at four different stages in the visual pathway of cats, were affected by aging. We found that: (1) there was a progressively greater effect of aging on neurons at successively higher hierarchical stages in the visual pathway. The contrast sensitivity of PMLS neurons had the most severe age-related deterioration, followed by A18 and A17, while LGN neurons were relatively unaffected by aging; and (2) reduced contrast sensitivity of neurons in three cortical areas was accompanied by enhanced maximal visually-evoked responses, increased spontaneous activity, and decreased SNR values, while the LGN was relatively unaffected by aging. Our findings suggest possible neural mechanisms underlying deficits on both easy and complex visual tasks observed during senescence (Habak and Faubert, 2000; Tang and Zhou, 2009).

The *C*_50_ values, acting as an index of the contrast sensitivity of neurons (Albrecht and Hamilton, 1982; Sclar et al., 1990; Yang et al., 2008), were differentially affected by aging in the four visual areas. The various areas along the visual pathway involve the processing of basic and complex perceptual functions, with more complex functions carried out at progressively higher levels (Van Essen et al., 1992; Elston and Rosa, 1997; Brewer et al., 2002). Those functions degrade during normal aging, but higher level functions are much more susceptible to aging (Habak and Faubert, 2000; Tang and Zhou, 2009). The functional degeneration in the old cat may reflect changes in the visual cortex (Crassini et al., 1988; Pardhan et al., 1996; Bennett et al., 1999). Our results are consistent with the previous findings reported in cats and macaque monkeys (Yang et al., 2008; Zhou et al., 2011) that there is an age-related impairment of contrast sensitivity of neurons in visual cortical cells, which may contribute, at least in part, to visual contrast sensitivity decline in aging. Furthermore, the current results also directly suggest that the contrast sensitivity of neurons in higher levels of the visual pathway is more vulnerable to aging, which could provide physiological evidence for the explanation of age-related deterioration for various visual tasks.

The present results demonstrated that the contrast response properties of LGN neurons were relatively unchanged in old cats, which was in accord with previous results of LGN in monkeys (Spear, 1993). However, the intact LGN response properties exclude the possibility that the degeneration of the visual cortex observed in the old cats, came from the deterioration of the optical system. In view of the age-related decline in the cortex described above, we hypothesize that the age-related changes would begin at the cortical level.

The neural circuitry at the cortical level is more complex than in LGN. While the visual information processing of X and Y cells is still well segregated, there is already substantial excitatory convergence in cortical areas A17, A18, and PMLS (Burke et al., 1998). In the following we consider how the age-related degeneration of contrast response properties of neurons is related to the complexity of neural circuitry, information convergence, and processing.

The function of higher-level neurons may be more vulnerable to aging than neurons at lower levels, due to the complexity of neural circuitry. Many neurophysiological and behavioral experiments suggested that the responses of neurons in higher cortical visual areas encode increasingly complex features of stimuli (Maunsell and Newsome, 1987; DeYoe and Van Essen, 1988), which might reasonably involve more complicated neural circuitry. Indeed, based on a series of studies on the effects of aging on perceptual processing and visual working memory capacity, Faubert (2002) argued that the decline in age-related perceptual abilities depended upon the complexity of the neural circuitry required for processing in a given task. The decline of contrast sensitivity of PMLS neurons was more severe than in A17 and A18, which suggested that the intracortical processing in PMLS could be affected more than in A17 and A18, by aging.

In addition to the complexity of neural circuitry, cumulative effects during information convergence across successive levels of neural processing may be an explanation underlying our findings. The visual system is organized hierarchically between areas (Heath and Jones, 1971). It has been hypothesized that cortical visual areas receive, process, and transmit visual information in a step wise manner (Symonds and Rosenquist, 1984). At each stage along the visual pathway, neurons receive information from lower levels, integrate and pool the information, and send it to higher levels. Thus, at successively higher stages along the visual hierarchy, visual responses are dependent upon ever greater amounts of preceding neural circuitry, each with its own susceptibility to age-related impairment. Consistently, in our results the effect of aging on A18 neurons was not significantly larger than on A17 neurons, however, the difference of *C*_50_ between young and old cats in A18 was moderately larger than A17. Both areas A17 and A18 in the cat receive their principal thalamic input from the LGN (Tretter et al., 1975; Raczkowski and Rosenquist, 1983; Sherk, 1986; Lomber et al., 1995), but A18 is not absolutely identical to A17. There are direct excitatory inputs to A18 from A17 (Burke et al., 1998).

Inhibitory circuits are essential components of the neural circuitry underlying various brain functions. Contrast normalization is thought to be mediated by intracortical suppression (Movshon et al., 1978b; DeBruyn and Bonds, 1986; Heeger, 1992; Carandini et al., 1997; Britten and Heuer, 1999). Our analysis of contrast response properties of cortical cells in young and old cats showed that old neurons exhibited decreased contrast sensitivity compared with young neurons. Previous studies have shown that GABA and its agonists improve visual cortical function in senescent monkeys (Leventhal et al., 2003). Therefore, the age differences in contrast normalization, observed in the current study, might be linked to a degradation of inhibitory intracortical circuits in old brain GABAergic and cholinergic pathways, that may have inhibitory effects in the visual cortex. Previous studies have hypothesized that the degeneration of inhibitory systems during aging may be an important mechanism underlying the age-related functional alterations (Leventhal et al., 2003; Hua et al., 2006; Wang et al., 2006; Bennett et al., 2007; Hua et al., 2008; Zhang et al., 2008). In our results, we did observe that the decreased contrast sensitivity of neurons was accompanied by an increased spontaneous activity, elevated visual evoked response, and decreased SNR values in old cats, all of which were consistent with a compromised intracortical inhibition during aging.

Our analyses of contrast response properties of X and Y cells showed that the *C_H_* values of X cells were slightly higher than that of Y cells, both in the young and the old group, but there was no significant difference. Additionally, the remaining contrast response properties (*R*_max_ and *M*) between X and Y cells also exhibited a similar trend. It was reported that Y cells were more sensitive to contrast than X cells (Enroth-Cugell and Robson, 1966). This disagreement may be caused by the small amount of sampled neurons in our experiments. Simple and complex cells in A17 and A18 did not differ in their contrast response properties in each group, although simple cells were, on average, slightly less sensitive to contrast than complex cells. These results were consistent with the observation of Albrecht and Hamilton (1982), that the difference of contrast response properties between simple and complex cells are, in general, quite small in the cat.

In summary, this study has demonstrated a progressively greater effect of aging on contrast sensitivity of neurons at successively higher stages of the visual pathway. The more severe impairment of aging neurons at higher levels could provide a potential explanation for the more pronounced deficits of complex visual functions during normal aging. The major goal of this investigation was to describe and characterize the effects of aging on the neurons at different stages in the visual pathway of the cat. Detailed information about effects on various cell types within each visual area will require further studies.

## Author contributions

Research and study design were carried out by all authors. The measurements were carried out by Zhengchun Wang, Zhimo Yao, and Nini Yuan. Zhengchun Wang contributed to the data analysis and to the manuscript writing.

## Conflict of interest statement

The authors declare that the research was conducted in the absence of any commercial or financial relationships that could be construed as a potential conflict of interest.
